# Accelerometric estimates of physical activity vary unstably with data handling

**DOI:** 10.1371/journal.pone.0187706

**Published:** 2017-11-06

**Authors:** Maia P. Smith, Marie Standl, Joachim Heinrich, Holger Schulz

**Affiliations:** 1 Institute of Epidemiology 1, Helmholtz Zentrum München—German Research Center for Environmental Health, Neuherberg, Germany; 2 Department of Public Health, School of Medicine, St George's University, Grenada, West Indies; 3 Institute and Outpatient Clinic for Occupational, Social and Environmental Medicine, Inner City Clinic, University Hospital of Munich (LMU), Munich, Germany; 4 CPC-Munich, Member of German Center for Lung Research, Munich, Germany; Vanderbilt University, UNITED STATES

## Abstract

**Background:**

Because of unreliable self-report, accelerometry is increasingly used to objectively monitor physical activity (PA). However, results of accelerometric studies vary depending on the chosen cutpoints between activity intensities. Population-specific activity patterns likely affect the size of these differences. To establish their size and stability we apply three sets of cutpoints, including two calibrated to a single reference, to our accelerometric data and compare PA estimates.

**Methods:**

1402 German adolescents from the GINIplus and LISAplus cohorts wore triaxial accelerometers (Actigraph GT3x) for one week (mean 6.23 days, 14.7 hours per day) at the hip. After validation of wear, we applied three sets of cutpoints for youth, including the most common standard (Freedson, 2005) and two calibrated to a single reference, (Romanzini uni- and triaxial, from Romanzini, 2014) to these data, estimating daily sedentary, light, moderate, vigorous and moderate-to-vigorous PA (MPA, VPA, MVPA). Stability of differences was assessed by comparing Romanzini’s two sets of cutpoints.

**Results:**

Relative agreement between cutpoints was closer for activity of lower intensities (largest difference for sedentary behaviour 9%) but increased for higher intensities (largest difference for light activity 40%, MPA 102%, VPA 88%; all p<0.01). Romanzini’s uniaxial and triaxial cutpoints agreed no more closely with each other than with Freedson’s.

**Conclusions:**

Estimated PA differed significantly between different sets of cutpoints, even when those cutpoints agreed perfectly on another dataset (i.e. Romanzini’s.) This suggests that the detected differences in estimated PA depend on population-specific activity patterns, which cannot be easily corrected for: converting activity estimates from one set of cutpoints to another may require access to raw data. This limits the utility of accelerometry for comparing populations in place and time. We suggest that accelerometric research adopt a standard for data processing, and apply and present the results of this standard in addition to those from any other method.

## Background

Physical activity (PA) is a major protective factor for most noncommunicable diseases [1, 2] and it is generally accepted that most populations in the developed world are insufficiently active.[3] However, even within a given population both estimates of PA levels [4] and changes over time [5, 6, 7] vary and thus interventions are difficult to design.

Because accelerometry is not vulnerable to reporting bias as are self-reports, it is an increasingly popular technique for assessing PA under field conditions. Accelerometers measure accelerations, which are then converted to unitless counts which are summed per unit time (epoch). Activity intensity (sedentary, light, moderate, or vigorous) in each epoch is defined by applying cutpoints that have been observed, in calibration studies, to best indicate metabolic demands [8, 9] in subjects of the appropriate age. However, these cutpoints vary substantially [4, 9, 10, 11, 12, 13] and thus accelerometrically-estimated PA in the same or similar population varies [4] based on the calibration study chosen. Furthermore, since different cutpoints were calibrated for different activities, the magnitude of differences between them may also vary depending on the pattern of body movement most common in the population. [14] This is especially true if one cutpoint measures only the vertical axis (uniaxial, as is typical of older studies) and the other measures all three axes (triaxial.) Thus accelerometric studies are difficult to compare to each other, and establishment of longitudinal, population-level differences in accelerometrically-estimated PA may be impossible.

Comparisons of different accelerometric protocols (cutpoints, weartime, epoch length) and device types have become increasingly common as accelerometry moves into the mainstream of research. [12, 14, 15, 16, 17, 18] These comparisons find large differences in estimated activity, but have not established the stability of these differences: for example, while Banda et al [15] found that the cutpoints of Evenson estimated 92% more MVPA than those of Treuth, they did not establish whether the difference is similar in other populations. If it is (i.e. the difference is stable) interconversion of activity estimates may be as simple as multiplication or division by a constant: while if it is not, interconversion may be impossible.

This study specifically addresses these concerns by applying three sets of cutpoints to the same dataset with the same data-cleaning protocol and comparing their estimates of activity in each intensity, quantified both as minute-by-minute agreement and as estimated total minutes. One set of cutpoints, created by Freedson et al, [9] is among the most common for youth; thus we apply it as a standard against which others should be compared. The other two (one uniaxial, one triaxial) were calibrated to a single test dataset by Romanzini et al[8] and thus agreed perfectly on activity intensity within it: thus any differences we observe appear to be specific to our population.

## Methods

### Study population

Both the GINIplus and LISAplus studies were approved by local Ethics Committees of Bavaria and West-Rhine Westphalia, and by written consent from participating families. This study sampled adolescents from two different population-based German birth cohorts: GINIplus and LISAplus, born between 1995 and 1999 in the regions of Munich and Wesel. Accelerometry was done between 2011 and 2014, and subjects were 15.6 (SD 0.5) years old at the time of accelerometry. Details on study design and cohort selection are published elsewhere. [19] [20] [21] We do not have the approval of the ethics committee nor of the subjects to make the data publicly available, but they are available to interested researchers who obtain approval of the GINIplus study steering committee (contact: Dr. Marie Standl, marie.standl@helmholtz-muenchen.de) and the ethics committees and acceptance of a data transfer agreement from the legal department of the Helmholtz Zentrum München.

Accelerometry participants were recruited from the entire 15-year followup of GINIplus and LISAplus that lived in Munich and Wesel, which is all of GINIplus but only 64% of LISAplus, since those LISAplus participants living in the study centers Bad Honnef and Leipzig were not approached for accelerometry. For more details on followup see [22] and [21]. Of the 3199 subjects from GINIplus who were successfully recontacted at age 15, all were approached for accelerometry, 1890 (59%) gave initial consent and 1247 (66%) gave final consent, completed successfully, and returned the device. Of 1107 LISAplus subjects who were from Munich or Wesel and thus approached for accelerometry, 654 (59%) gave initial consent and 435 completed (66%). Of the 1682 adolescents from GINIplus and LISAplus who completed accelerometry, 1411 (83%) successfully passed data-quality checks and 1402 (33% of those initially approached for accelerometry) wore a device that captured triaxial acceleration. These 1402 are included in the current study.

### Accelerometry protocol

Accelerometry protocol has been described at [23]. Subjects wore triaxial accelerometers (ActiGraph GT3X, Pensacola, Florida) on the dominant hip for 7 consecutive days, after which they were returned by mail. Subjects documented each of the following events as close as possible to the time they occurred: time of waking up and going to bed; time and reason for removing the monitor (non-wear time, NWT) such as for showering or swimming; time and method of travel to school; time of starting and finishing school; time of starting and finishing school sport; and time and type of leisure sporting activity. Diary information was digitized using a 7-day template and reviewed by a second study assistant to avoid transcription errors. The current study considered only time spent during diaried waking time, which was filtered out of the 24-hour signal on a day-to-day basis for each subject based on time between reported “getting up” and “going to bed”. Since the goal of this study was to capture a representative sample of daily PA, days were disqualified if they were judged to be not representative of typical routine, such as days spent sick or travelling: more details are given in [24] and [25]. NWT was identified by comparing the diary data to the results from the monitor according to the NWT algorithm of Troiano [26] using SAS programs published by NHANES[27] and by visual inspection of accelerometer tracings in case of discrepancy with the diary. In most cases the diary agreed with the automatic programs upon wear time and NWT: of the recorded 11,572 days of accelerometer wear, 9.85% (1140) had to be visually inspected due to differences between diary and algorithm. Days were excluded if the discrepancy was larger than the 10^th^ and 90^th^ percentiles in a subset of this cohort:[24] more than 45 minutes of diaried NWT when the program indicated the device was worn, or more than 150 minutes where the program indicated NWT but the diary did not. During these short periods of discrepancy, the diary data was used.

Sampling rate was set to 30 Hz and the measured accelerations stored at 1 Hz after conversion into activity counts. Data were summed over 60-second epochs. Data filtering was set to default (‘normal’) as recommended by ActiGraph. ActiLife software was used for initialization of accelerometers (version 5.5.5, firmware 4.4.0) and for download of data. The 60-second epoch length chosen was the most common in two recent reviews: Cain et al [28] found that of 68 accelerometric studies in adolescents, over half (63%) used an epoch length of 60 seconds, 13% used 30 seconds, 4.4% 15 seconds and 3% less than 5 seconds, and Guinhoya et al [4] found that 60-second epochs were the commonest even when including studies of children, as well as adolescents. Furthermore, it is known from sport physiology [29] that cardiovascular adaptation to a certain exercise level takes about 1 to 2 minutes before reaching a steady state. Thus the physiological benefits of very brief epochs of PA have not been ascertained, and our choice of 60 seconds represents both clinical relevance and maximal intercomparability with other studies.

Activity counts per minute when the subject was awake were assigned to the four intensity levels—sedentary, light, moderate, and vigorous physical activity—using Freedson’s (2005) commonly-used uniaxial cut-points for children [9] for the vertical axis, and Romanzini’s uniaxial and triaxial cutpoints for adolescents[8] for the vertical axis and the triaxial signal, respectively. These cutpoints are given in S3 Table.

### Statistical methods

All statistical analyses used SAS 9.2. All graphics were created using Excel. All analyses were limited to validated recording time where the subject reported being awake and out of bed.

Mean minutes in each activity intensity for each subject, according to each cutpoint, was calculated and then summarized across subjects. These summary results are presented as mean (standard deviation), 5^th^, 95^th^ percentiles. To establish the significance of differences, linear or log-linear models were fit to compare each pair of algorithms’ estimate of number of minutes in activity of each intensity. Only the largest (i.e. least significant) p-value is shown. These differences are then visually presented using Bland-Altman plots. [30] Romanzini’s two cutpoints are compared in the main text; each is compared to Freedson’s in S1 Table, S2 Fig and S1 Fig.

Interalgorithm agreement on the intensity of each minute’s activity is presented using cross-tabulations, without statistical tests. Each minute was classified into the four intensities by each of Romanzini’s cutpoints, and we present the percent of minutes that fell into each possible combination of estimated intensities. Romanzini’s cutpoints are compared to Freedson’s in S1 Fig and S2 Fig.

## Results

Daily activity for 1402 Germans (mean age 15.6 years, 46% male) was accelerometrically monitored over 4–7 days per subject for an average of 14.7 hours per day. (Table 1) Detailed population characteristics and activity levels according to Freedson’s cutpoints have been previously published.[25] [31] All three sets of cutpoints agreed that subjects spent about 2/3 of time sedentary (range: 67–74%); and about 5% of time in MVPA (range: 4.06–5.67) with the remainder in light activity (LPA.) (Table 1, Table 2, S1 Table and S2 Table). However, estimated MPA and VPA differed by over 50% between cutpoints and all pairwise comparisons between cutpoints’ estimates of activity minutes of each intensity were significant at p = 0.01. All but one (MPA, Romanzini triaxial vs Freedson) were also significant at p<0.0001. (Table 1.)

**Table 1:** Population characteristics. Mean (standard deviation) unless otherwise stated.

		P-value for pairwise difference between cutpoints
N	1402	—
Male (N, %)	650, 46	—
Age, years	15.6 (0.5)	—
Height, cm	172 (8.2)	—
Weight, kg	61.6 (11)	—
BMI, kg/m^2^	20.8 (3.0)	—
BMI Z-score^1^	0.10 (0.97)	
Parents highly educated^2^, %	71	—
Days of accelerometry (range 4–7)	6.26 (0.88)	—
Accelerometric min/day	884 (51)	—
Sedentary behavior, min/day		All <0.0001
Freedson	591 (74)	
Romanzini uniaxial	645 (71)	
Romanzini triaxial	**651 (72)**	
Light activity, min/day		All <0.0001
Freedson	**253 (56)**	
Romanzini uniaxial	203 (50)	
Romanzini triaxial	183 (44)	
Moderate activity, min/day		0.0083 for Freedson vs. Romanzini triaxial: <0.0001 for others
Freedson	**28.7 (15)**	
Romanzini uniaxial	14.2 (7.6)	
Romanzini triaxial	27.7 (13)	
Vigorous activity, min/day		All <0.0001
Freedson	12.6 (12)	
Romanzini uniaxial	22.5 (16)	
Romanzini triaxial	**23.7 (19)**	
Moderate or vigorous activity, min/day		All <0.0001
Freedson	41.2 (23)	
Romanzini uniaxial	36.7 (22)	
Romanzini triaxial	**51.4 (29)**	

Largest estimate of activity for each intensity in **bold.**

1) BMI Z-scores from World Health Organization Child Growth Standards, Growth reference 5–19 years

2) Higher-educated parent entered college or higher. Very similar population profiled in (Smith et. al, 2016; PLOSOne. doi: 10.1371/journal.pone.0152217.)

P-values from generalized linear models. All pairwise comparisons between cutpoints checked.

**Table 2:** Agreement of cutpoints on activity intensity, minute by minute. Percent of time (total 14.7 hours / day, 8780 days).

		**Romanzini Triaxial**				
**Romanzini Uniaxial**		Sedentary	Light	Moderate	Vigorous	**Total**
	Sedentary	**70.38**	*2*.*66*	*<0*.*001*	*<0*.*001*	73.04
	Light	3.29	**17.93**	*1*.*52*	*0*.*16*	22.90
	Moderate	—	0.06	**1.16**	0.36	1.58
	Vigorous	—	—	0.41	**2.07**	2.48
	**Total**	73.67	20.65	3.08	2.59	100

**Bold text** for minutes in which Romanzini’s triaxial and uniaxial cutpoints estimate activity of the same intensity: italics for minutes where triaxial cutpoints estimate more-intense activity. Romanzini’s uniaxial and triaxial cutpoints calibrated to the same reference population in (Romanzini et. al, 2014).

–if no minutes fell into that category (e.g. sedentary according to the triaxial cutpoints, but vigorous according to uniaxial.)

The three cutpoints agreed well on total estimated sedentary behaviour (largest difference 9%, Table 1), but disagreed more, in relative terms, for activity of higher intensity. The largest pairwise differences were 40% for LPA, 102% for MPA, 88% for VPA, and 40% for MVPA. Romanzini’s triaxial cutpoints estimated the most sedentary behaviour, the least LPA, and the highest MVPA and VPA; their estimated MPA was close to that of Freedson. Romanzini’s triaxial cutpoints on average estimated 27.7 (SD = 13) minutes MPA and 23.7 (19) minutes VPA per day, or 51.4 minutes MVPA: 41% more than the uniaxial cutpoints calibrated to the same reference population which estimated 14.2 (SD 7.6) minutes MPA per day and 22.5 (16) minutes VPA. For Freedson the corresponding numbers were 28.7 (15) and 12.6 (12) minutes MPA and VPA: twice as much MPA and just over half as much VPA, with total MVPA 36.7 and 41.3 minutes respectively.

Table 2 compares estimated activity intensity minute-by-minute for Romanzini’s two sets of cutpoints. Freedson’s cutpoints are compared to both of Romanzini’s in S1 Table and S2 Table. Although for most minutes the two sets of cutpoints agree on activity intensity, some scatter is visible: in particular the triaxial cutpoints estimate much higher activity than the uniaxial ones for a few minutes. The uniaxial cutpoints estimated that 73% of time was sedentary, 23% in LPA, 1.6% in MPA, and 2.5% in VPA: corresponding numbers for triaxial were 74%, 21%, 3.1%, and 2.6%. Of all the minutes classified by triaxial accelerometry as MPA, a few were classified by the uniaxial cutpoints as sedentary; almost half as LPA; and 13% as VPA. Of all the minutes classified by uniaxial accelerometry to be MPA, the triaxial signal indicated that none were sedentary, 4% were LPA, 6.6% were MPA, and 23% were vigorous.

**Fig 1:**
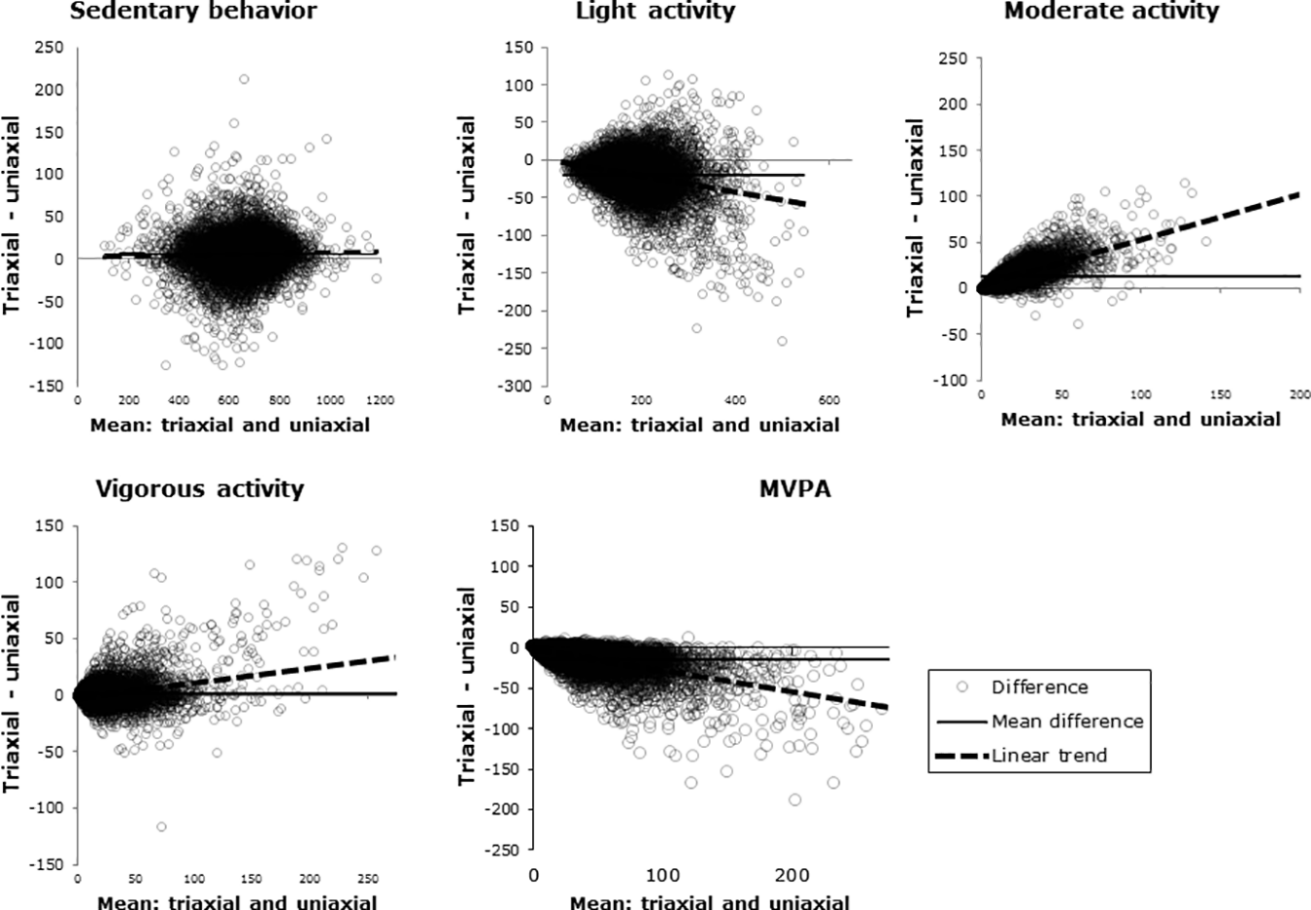
Large differences in estimated activity intensity between uni- and triaxial accelerometric cutpoints originally calibrated to the same dataset. Romanzini’s uni- and triaxial cutpoints from Romanzini M; Petroski, EL; Ohara, D; Dourado, AC; Reichert, FF. Calibration of ActiGraph GT3X, Actical and RT3 accelerometers in adolescents. European Journal of Sport Science. 2014;14(1):91–9. doi: 10.1080/17461391.2012.732614.

In addition to the above-described fixed bias, there was also proportional bias for most activity intensities. In Bland-Altman plots (Fig 1, “Activity minutes by two sets of cutpoints“) the difference between estimated minutes in each activity intensity, according to Romanzini’s uni- and triaxial cutpoints, is plotted against the mean of the two for each recording day. The thin solid line (“Mean difference”) shows the average difference for all recording days; the dotted black line (“Linear trend”) shows how this difference varies as a function of activity level (amount of time in that intensity.) Proportional bias is obvious for the higher activity intensities: as more time is spent in each intensity, the mean difference between the two algorithms gets further from zero. For LPA and MVPA, the difference becomes more and more negative; while for MPA and VPA, the difference becomes more and more positive. Only sedentary time had little proportional bias, and still estimated sedentary time varied widely between cutpoints: 5^th^ and 95^th^ percentiles of the difference were -23 and 314 minutes per day.

## Discussion

Although accelerometry is an objective measure of acceleration, it is not an objective measure of either physical activity or energy expenditure.[32] Furthermore, differences in data handling have potential to affect accelerometric estimates of activity above and beyond limitations of the method itself. In this large study of adolescents, we empirically confirm [4, 17, 26] large differences in accelerometrically-estimated physical activity depending on the cutpoints chosen. We add that accelerometric calibration algorithms which agree on one dataset may still disagree on another, and thus population-specific features may contribute to differences in estimated PA between algorithms. Converting estimated activity according to one set of cutpoints into another appears to be impossible without access to the raw data: since two sets of cutpoints can agree on one dataset and disagree on another, simple multiplication or division of estimates is not sufficient.

Ours is not the first study to find large differences in estimated accelerometric physical activity depending on data handling. [4, 9, 10, 11, 12, 13, 14, 15, 16, 17, 18] However, we are among the first to prove the effect of cutpoints alone and to prove that differences in estimated activity level by cutpoint are themselves not fixed. While a recent review [4] found even larger differences than we did, these are from different studies and thus could conceivably be attributed to weartime protocols, device type, or even population differences. The same is true for most studies which examine the effects of data handling, in which weartime protocols, epoch length, and cutpoints may all simultaneously vary.

While it is often suggested [10] that triaxial accelerometry differs significantly from uniaxial for capturing activities of daily living, comparatively few studies (with the exception of Vanhelst) [14] have explicitly compared the two as done in our study. Vanhelst found close agreement between uni- and triaxial accelerometric estimates of moderate and vigorous physical activity. In contrast, we found that relative differences were larger for high activity intensities and smaller for sedentary behavior. Vanhelst’s population and data-handling protocol were similar to ours, including epoch length and device weartime: however, the biggest differences are likely to be in the cutpoints chosen. Theirs appeared to be from two different studies, which in turn were almost certainly calibrated to different activities: the uniaxial cutpoints between activity levels were often higher than the triaxial ones. [14] In contrast, Romanzini’s uni- and triaxial cutoffs are calibrated to the same test dataset and thus triaxial counts cannot be less than uniaxial. However, we concur with Vanhelst that there was no large difference between uni- and triaxial estimates of activity in daily living: indeed, in our study two different uniaxial sets of cutpoints were about as similar as each was to a set of triaxial cutpoints. It is plausible that differences are larger during activities with primarily non-vertical patterns of acceleration, such as certain sports: however sport was a relatively small contributor to total physical activity in this sample. [23] Thus we concur with the literature that triaxial accelerometry was generally comparable to uniaxial in monitoring activities of daily living: the effect of the additional two movement axes was small compared to that of cutpoints.

However, our study of the effects of data handling on activity estimates is not immune to the effects we describe. While we describe the decisions we made for each of many data-handling protocols, we recognize that other decisions would have been almost equally valid and consistent with the literature. For example, while a 60-second epoch length is the commonest in this age group [28] it is also the longest and longer than that used to validate two of the cutpoints[8]. This creates a discrepancy which may [28] contribute to systematic over- or underestimates of MPA and/or VPA in our study; however Banda et al found ([33]) that Romanzini’s cutpoints estimated similar levels (within 25%) of MVPA whether 15-second or 60-second epochs were used.

Similar caveats apply to decisions to accept data from a given subject or day of recording, and to criteria for identifying NWT. Since NWT is most likely to be confused with sedentary time, falsely identifying NWT as sedentary time may artificially inflate weartime, lead to the acceptance of time and days where in fact the device was not worn, and thus underestimate total daily activity. For this reason, our estimates of total physical activity should only be compared to other studies using similar devices and data-handling protocols.

Our findings of large and variable differences between accelerometric cutpoints add to the growing scientific consensus [4, 9, 10, 11, 12, 13, 14, 15, 16, 17, 18] that accelerometry, as a method, cannot be viewed as objective if data handling is not standardized. The need for standardization is particularly acute as accelerometric research expands from the academic to the private sector. Data are collected with wearable devices such as smartphones, processed with apps, and combined in real time with data from other apps which already track sleep, detect sleep apnea, [34]or detect eating.[35] Activity trackers are easier to use than dedicated accelerometers, are much less expensive, and can even provide real-time feedback for desired or undesired behaviors, such as eating or physical activity. [36, 37] Traditional methods of accelerometry cannot compete with this rapid growth and diversification: scientific consensus cannot keep up with commercial innovation.

However, the flexibility of activity-tracking apps is also their greatest weakness: since their data collection and processing constantly changes they cannot be used to establish records of past physical activity [36] and thus they are unlikely to be useful for any attempt to establish population-level changes in activity pattern. In contrast, cutpoint-based accelerometry is replicable and consistent: it has existed for over thirty years [38] compared to ten for the iPhone, and data-handling protocols used in scientific studies are publicly available. Both academic and non-academic methods of accelerometric activity assessment have distinct strengths, which could be profitably combined to benefit both researchers and the general public.

Validation studies show close agreement between activity counts registered by Actigraph accelerometers and both smartphones[39, 40, 41] and other devices (e.g. Fitbit, Jawbone)[39] in activities of daily living, which suggests that the hardware of these devices is adequate to capture physical activity if the phone is carried in a pocket or the device is worn as directed. Thus apps could be designed to apply a validated data-handling protocol (standards for epoch length, detection of NWT, cutpoints between intensities, and total weartime) using the sensors in these devices. [39]Since data processing may not be of primary interest to physical-activity researchers, we believe that many would prefer to collect data with a peer-reviewed and validated app rather than dealing with an expensive dedicated device, proprietary software, and complex data-handling protocols which often offer no clear guidance. [28] Activity estimates based on a standard protocol could grow to play the same role in population-level activity assessment as does body mass index in estimating adiposity: as a replicable standard which, in spite of known limitations, provides useful estimates of population-level trends.

Use of a single standard protocol does not preclude improvements in methodology. Data processing and transmission are now inexpensive enough to allow multiple algorithms to be applied to the same dataset (as is done here and elsewhere[15]) and published either in the main text or online in a supplement: soon even raw data will be archivable and thus available for reprocessing with the newest algorithms. We encourage researchers (academic and private) to use up-to-date methods of handling and processing data: however we suggest that they do this in addition to, not instead of, creating replicable standards which ensure compatibility with past and future research.

## Conclusions

In a single dataset, we found that adolescents’ estimated moderate and vigorous physical activity differed by up to 50% based on the cutpoints used for data handling. This difference was highly statistically significant and would be clinically relevant if it were real and not artefactual. Furthermore agreement was no better with a pair of algorithms calibrated to the same test dataset [8] than with two algorithms calibrated to different test datasets [8, 9]: thus differences appear to themselves vary with population. It may be impossible to convert activity estimates from one algorithm to another if raw data are not available, and this limits the utility of accelerometry as a research technique. Thus we suggest that academic accelerometric research be standardized, with all studies of each age group publishing estimates processed in a standard way in addition to more modern methods of data processing.

## Supporting information

S1 Table Large differences in estimated activity intensity minute by minute from two sets of uniaxial cutpoints. Percent of time (total 14.7 hours / day, 8780 days). If no minutes fell into that category (e.g. sedentary according to one set of cutpoints, but vigorous according to the other.)
(DOC)

S2 Table Large differences in estimated activity intensity minute by minute from two sets of cutpoints. Percent of time (total 14.7 hours / day, 8780 days).
If no minutes fell into that category (e.g. sedentary according to one set of cutpoints, but vigorous according to the other.).
Freedson’s algorithm for children from Freedson P; Pober, D; Janz, KF Calibration of accelerometer output for children. Med Sci Sports Exerc. 2005;37(11(Suppl)):523–30. Romanzini’s triaxial algorithm from Romanzini M; Petroski, EL; Ohara, D; Dourado, AC; Reichert, FF. Calibration of ActiGraph GT3X, Actical and RT3 accelerometers in adolescents. European Journal of Sport Science. 2014;14(1):91–9. 10.1080/17461391.2012.732614.
(DOC)

S3 Table Accelerometric cutpoints. Counts per minute (bottom of category).
Freedson’s cutpoints from Freedson P; Pober, D; Janz, KF Calibration of accelerometer output for children. Med Sci Sports Exerc. 2005;37(11(Suppl)):523–30. Romanzini’s cutpoints from Romanzini M; Petroski, EL; Ohara, D; Dourado, AC; Reichert, FF. Calibration of ActiGraph GT3X, Actical and RT3 accelerometers in adolescents. European Journal of Sport Science. 2014;14(1):91–9.
(DOC)

S1 Fig Large differences in estimated activity intensity minute by minute by two sets of accelerometric cutpoints. Freedson’s uniaxial (vertical) algorithm for children from Freedson P; Pober, D; Janz, KF Calibration of accelerometer output for children. Med Sci Sports Exerc. 2005;37(11(Suppl)):523–30.
Romanzini’s triaxial algorithm from Romanzini M; Petroski, EL; Ohara, D; Dourado, AC; Reichert, FF. Calibration of ActiGraph GT3X, Actical and RT3 accelerometers in adolescents. European Journal of Sport Science. 2014;14(1):91–9. 10.1080/17461391.2012.732614.
(TIF)

S2 Fig Large differences in estimated activity intensity minute by minute by two sets of accelerometric cutpoints. Freedson’s uniaxial (vertical) algorithm for children from Freedson P; Pober, D; Janz, KF Calibration of accelerometer output for children. Med Sci Sports Exerc. 2005;37(11(Suppl)):523–30.
Romanzini’s uniaxial (vertical) algorithm from Romanzini M; Petroski, EL; Ohara, D; Dourado, AC; Reichert, FF. Calibration of ActiGraph GT3X, Actical and RT3 accelerometers in adolescents. European Journal of Sport Science. 2014;14(1):91–9. 10.1080/17461391.2012.732614.
(TIF)

## Abbreviations

LPA: light physical activity
MPA: moderate physical activity
MVPA: moderate-to-vigorous physical activity
NWT: (accelerometer) non-wear time
PA: physical activity
VPA: vigorous physical activity
