# Supplementary material for: Accelerometric estimates of physical activity vary unstably with data handling
Source: PLoS One. 2017 Nov 6;12(11):e0187706. doi: 10.1371/journal.pone.0187706 (PMC5673210; doi:10.1371/journal.pone.0187706)
Supplement: S1 Table — Percent of time (total 14.7 hours / day, 8780 days). If no minutes fell into that category (e.g. sedentary according to one set of cutpoints, but vigorous according to the other.) (DOC) [file pone.0187706.s001.doc]

|  |  | **Romanzini Uniaxial** | | | |  |
| --- | --- | --- | --- | --- | --- | --- |
| **Freedson** |  | Sedentary | Light | Moderate | Vigorous | **Total** |
|  | Sedentary | **66.91** | -- | -- | -- | 66.91 |
|  | Light | 6.13 | **22.41** | -- | -- | 28.54 |
|  | Moderate | -- | 0.50 | **1.58** | 1.10 | 3.18 |
|  | Vigorous | -- | -- | -- | **1.37** | 1.37 |
|  | **Total** | 73.04 | 22.91 | 1.58 | 2.48 | 100 |
