# Supplementary material for: Accelerometric estimates of physical activity vary unstably with data handling
Source: PLoS One. 2017 Nov 6;12(11):e0187706. doi: 10.1371/journal.pone.0187706 (PMC5673210; doi:10.1371/journal.pone.0187706)
Supplement: S2 Table — Percent of time (total 14.7 hours / day, 8780 days). If no minutes fell into that category (e.g. sedentary according to one set of cutpoints, but vigorous according to the other.). Freedson’s algorithm for children from Freedson P; Pober, D; Janz, KF Calibration of accelerometer output for children. Med Sci Sports Exerc. 2005;37(11(Suppl)):523–30. Romanzini’s triaxial algorithm from Romanzini M; Petroski, EL; Ohara, D; Dourado, AC; Reichert, FF. Calibration of ActiGraph GT3X, Actical and RT3 accelerometers in adolescents. European Journal of Sport Science. 2014;14(1):91–9. 10.1080/17461391.2012.732614. (DOC) [file pone.0187706.s002.doc]

|  | | **Romanzini Triaxial** | | | |  |
| --- | --- | --- | --- | --- | --- | --- |
| **Freedson** |  | Sedentary | Light | Moderate | Vigorous | **Total** |
|  | Sedentary | **65.86** | 1.05 | <0.001 | <0.001 | 66.91 |
|  | Light | 7.81 | **19.41** | 1.20 | 0.11 | 28.54 |
|  | Moderate | -- | 0.20 | **1.88** | 1.11 | 3.18 |
|  | Vigorous | -- | -- | <0.001 | **1.37** | 1.37 |
|  | **Total** | 73.67 | 20.66 | 3.08 | 2.59 | 100 |
