# Supplementary material for: Accelerometric estimates of physical activity vary unstably with data handling
Source: PLoS One. 2017 Nov 6;12(11):e0187706. doi: 10.1371/journal.pone.0187706 (PMC5673210; doi:10.1371/journal.pone.0187706)
Supplement: S3 Table — Counts per minute (bottom of category). Freedson’s cutpoints from Freedson P; Pober, D; Janz, KF Calibration of accelerometer output for children. Med Sci Sports Exerc. 2005;37(11(Suppl)):523–30. Romanzini’s cutpoints from Romanzini M; Petroski, EL; Ohara, D; Dourado, AC; Reichert, FF. Calibration of ActiGraph GT3X, Actical and RT3 accelerometers in adolescents. European Journal of Sport Science. 2014;14(1):91–9. (DOC) [file pone.0187706.s003.doc]

|  | Sedentary | Light | Moderate | Vigorous |
| --- | --- | --- | --- | --- |
| Freedson | **0** | **100** | **2220** | **4136** |
| Romanzini uniaxial | **0** | **185** | **2427** | **3272** |
| Romanzini triaxial | **0** | **721** | **3028** | **4448** |
