# Supplementary figures and images for: Accelerometric estimates of physical activity vary unstably with data handling
Source: PLoS One. 2017 Nov 6;12(11):e0187706. doi: 10.1371/journal.pone.0187706 (PMC5673210; doi:10.1371/journal.pone.0187706)

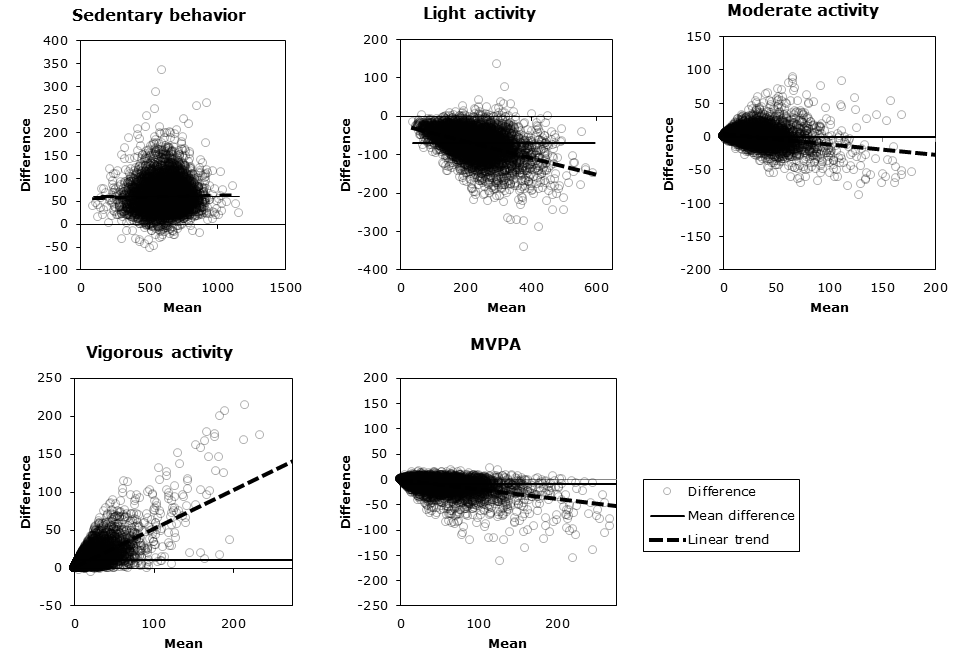

Supplement: S1 Fig — Freedson’s uniaxial (vertical) algorithm for children from Freedson P; Pober, D; Janz, KF Calibration of accelerometer output for children. Med Sci Sports Exerc. 2005;37(11(Suppl)):523–30. Romanzini’s triaxial algorithm from Romanzini M; Petroski, EL; Ohara, D; Dourado, AC; Reichert, FF. Calibration of ActiGraph GT3X, Actical and RT3 accelerometers in adolescents. European Journal of Sport Science. 2014;14(1):91–9. 10.1080/17461391.2012.732614. (TIF) [file pone.0187706.s004.tif]

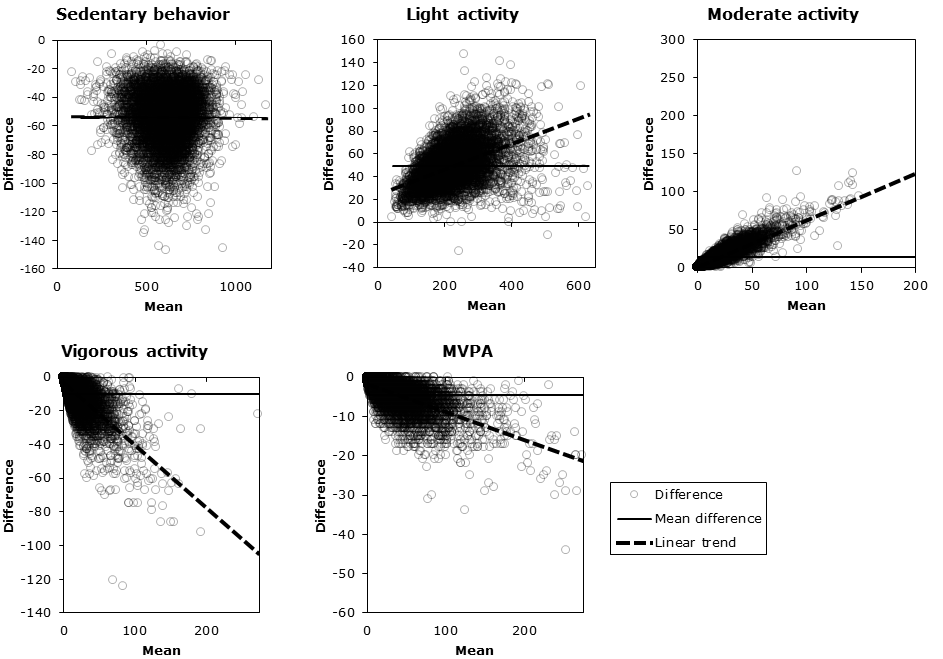

Supplement: S2 Fig — Freedson’s uniaxial (vertical) algorithm for children from Freedson P; Pober, D; Janz, KF Calibration of accelerometer output for children. Med Sci Sports Exerc. 2005;37(11(Suppl)):523–30. Romanzini’s uniaxial (vertical) algorithm from Romanzini M; Petroski, EL; Ohara, D; Dourado, AC; Reichert, FF. Calibration of ActiGraph GT3X, Actical and RT3 accelerometers in adolescents. European Journal of Sport Science. 2014;14(1):91–9. 10.1080/17461391.2012.732614. (TIF) [file pone.0187706.s005.tif]
